# Regeneration of Jaw Joint Cartilage in Adult Zebrafish

**DOI:** 10.3389/fcell.2021.777787

**Published:** 2022-01-20

**Authors:** Joanna Smeeton, Natasha Natarajan, Troy Anderson, Kuo-Chang Tseng, Peter Fabian, J. Gage Crump

**Affiliations:** ^1^ Department of Rehabilitation and Regenerative Medicine, Columbia Stem Cell Initiative, Columbia University Irving Medical Center, Columbia University, New York, NY, United States; ^2^ Department of Genetics and Development, Columbia Stem Cell Initiative, Columbia University Irving Medical Center, Columbia University, New York, NY, United States; ^3^ Department of Stem Cell Biology and Regenerative Medicine, Keck School of Medicine, University of Southern California, Los Angeles, CA, United States

**Keywords:** joint, osteoarthritis, zebrafish, cartilage, regeneration

## Abstract

The poor intrinsic repair capacity of mammalian joint cartilage likely contributes to the high incidence of arthritis worldwide. Adult zebrafish can regenerate many structures that show limited or no healing capacity in mammals, including the jawbone. To test whether zebrafish can also regenerate damaged joints, we developed a surgical injury model in which the zebrafish jaw joint is destabilized *via* transection of the major jaw joint ligament, the interopercular–mandibular (IOM). Unilateral transection of the IOM ligament in 1-year-old fish resulted in an initial reduction of jaw joint cartilage by 14 days, with full regeneration of joint cartilage by 28 days. Joint cartilage regeneration involves the re-entry of articular chondrocytes into the cell cycle and the upregulated expression of *sox10*, a marker of developing chondrocytes in the embryo that becomes restricted to a subset of joint chondrocytes in adults. Genetic ablation of these *sox10*-expressing chondrocytes shows that they are essential for joint cartilage regeneration. To uncover the potential source of new chondrocytes during joint regeneration, we performed single-cell RNA sequencing of the uninjured adult jaw joint and identified multiple skeletal, connective tissue, and fibroblast subtypes. In particular, we uncovered a joint-specific periosteal population expressing *coch* and *grem1a*, with the jaw joint chondrocytes marked by *grem1a* expression during regeneration. Our findings demonstrate the capacity of zebrafish to regenerate adult joint cartilage and identify candidate cell types that can be tested for their roles in regenerative response.

## Highlights

• Ligament transection induces cartilage loss at the adult zebrafish jaw joint
• Regeneration of joint cartilage within 1 month post-injury
• Requirement of *sox10*-expressing cells for joint cartilage regeneration
• Single-cell sequencing reveals the diversity of cell types within the adult jaw joint

## Introduction

Vertebrate synovial joints are complex structures composed of multiple integrated cell types, including osteoblasts, superficial and deeper chondrocytes, ligamentocytes, and connective tissue cells lining the synovium and adjacent periosteum of the underlying bone. In osteoarthritis, a progressive disease affecting joints, aging and/or injury results in loss of joint cartilage, erosion of underlying bone, synovial hyperplasia, osteophyte formation, chronic inflammation, and likely other changes to joint-associated cell types (Glasson et al., 2010). Osteoarthritis is considered a terminal disease in adult humans and mice, with little to no evidence that joint cartilage can recover after the onset of the disease. To understand the complex cellular changes underlying arthritis progression and the failure of resident cells to counteract joint degeneration, recent studies have focused on cataloging joint-resident cells in normal and diseased states (Ji et al., 2019; Bian et al., 2020; Sebastian et al., 2021). However, as these have been performed in largely non-regenerative mammals (primarily mouse and human), it has been difficult to define putative endogenous progenitor populations with latent capacity for joint repair. One exception is the temporomandibular jaw joint (TMJ) of mouse, an unusual joint with a fibrocartilaginous disc that has been shown to possess fibrocartilage stem cells that can form self-renewing colonies *in vitro* and repair the damaged TMJ *in vivo* (Embree et al., 2016; Bi et al., 2020).

We had previously shown that the joints of the zebrafish jaw and fin have synovial properties and are susceptible to degenerative arthritis in the absence of the lubricin-encoding gene *prg4b* (Askary et al., 2016), similar to the arthritis of synovial joints seen in mice and humans lacking lubricin (Rhee et al., 2005; Alazami et al., 2006; Hill et al., 2014; Koyama et al., 2014). Here we establish a novel acute injury model in the adult zebrafish jaw joint, with an aim toward understanding the cellular contributions to joint repair in a highly regenerative vertebrate. Common rodent models of osteoarthritis of the knee involve surgical destabilization of the medial meniscus (DMM) or transection of the anterior cruciate ligament (Fang and Beier, 2014). We therefore tested whether the zebrafish jaw joint would be susceptible to a similar destabilizing trauma. Unlike rodent surgical destabilization models, where ligaments fail to regenerate and irreversible arthritic changes ensue, we find that zebrafish can recover from destabilizing injuries that cause a transient loss of joint-lining cartilage. We then performed single-cell RNA sequencing of the uninjured zebrafish jaw joint to identify the diversity of cell populations in this regenerative joint. The establishment of a synovial joint regeneration model in zebrafish thus provides an opportunity to test the putative roles of distinct joint-resident populations in articular cartilage regeneration.

## Results

### Development of an Acute Joint Injury Model in Zebrafish

**FIGURE 1:**
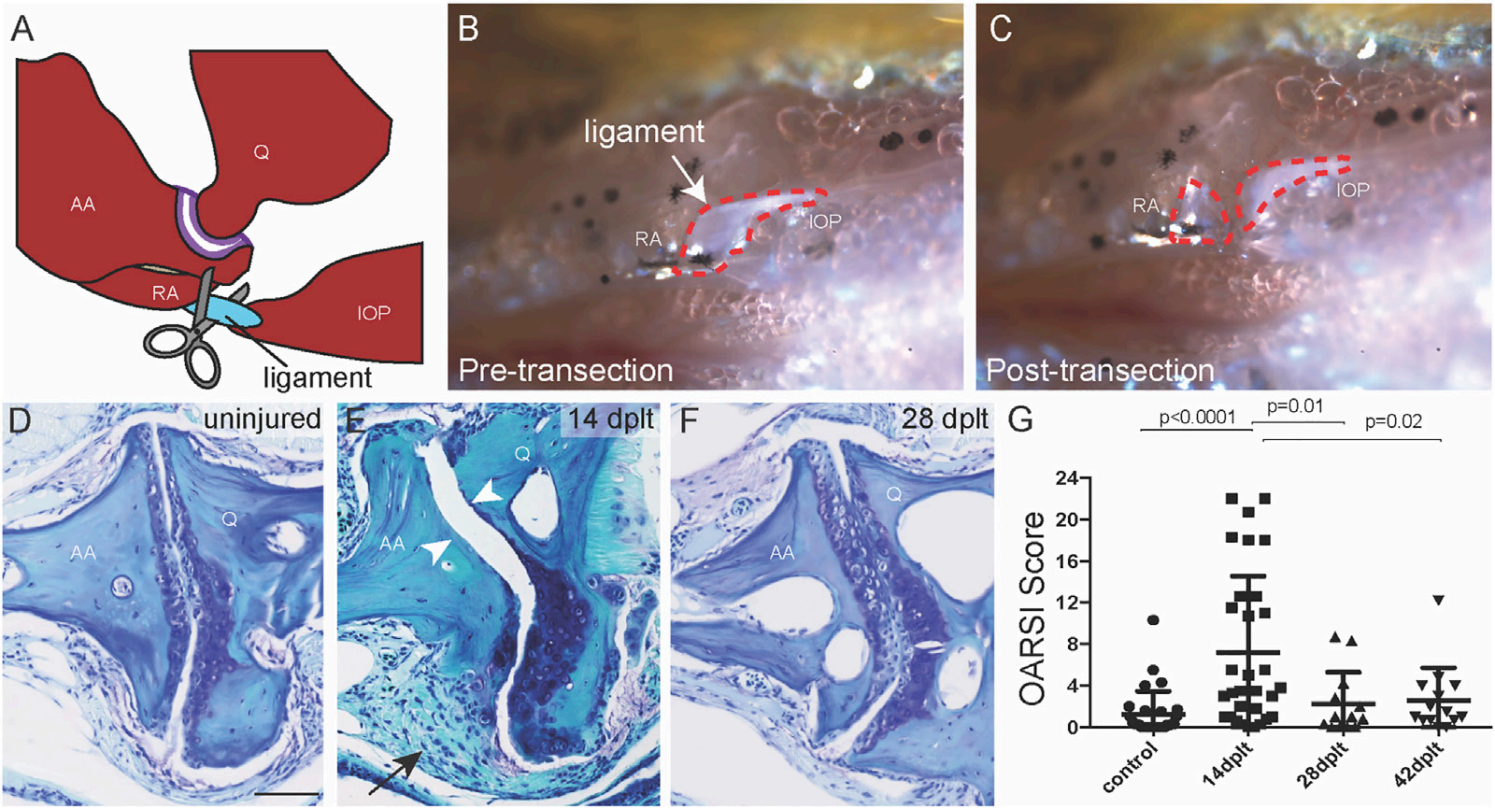
Joint degeneration and recovery following ligament transection surgery. **(A)** Schematic of the bones of the zebrafish jaw relative to the jaw joint (purple) and the interopercular–mandibular (IOM) ligament (blue) that is surgically transected. **(B,C)** Reflected light images of the zebrafish jaw in ventral view show the IOM ligament (dotted red outline) before **(B)** and immediately after **(C)** transection. **(D–F)** Toluidine blue staining of uninjured adult jaw joint **(D)** and IOM-transected jaw joint at 14 days post-ligament transection (dplt) **(E)** and 28 dplt **(F)**. The arrowheads point to the area of cartilage erosion and the arrow to the synovial hyperplasia. **(G)** Quantification of jaw joint degeneration after IOM transection using the zebrafish OARSI scoring method; *n* = 29 uninjured; *n* = 31 at 14 dplt; *n* = 13 at 28 dplt; *n* = 14 at 42 dplt. Individual data points, mean, and standard error of the mean are shown. The *p*-values are calculated by ANOVA and Tukey’s multiple-comparison test. AA, anguloarticular bone; IOP, interopercular bone; Q, quadrate bone; RA, retroarticular bone. Scale bar = 50 μm.

As destabilization of joint biomechanics due to ligament injury induces osteoarthritis in rodent models, we sought to establish a similar model in the zebrafish jaw joint. The IOM ligament connects the retroarticular bone, an outgrowth of the articulating mandibular bone of the jaw joint, with the more posterior interopercular that is part of a series of bones supporting the gill cover (Hu and Albertson, 2017). Among the complex series of mechanical couplings that control the movement of the jaw joint, the IOM ligament transmits the force generated by the opercular series of bones to open and close the lower jaw (Westneat et al., 2005). Using fine surgical scissors, we fully cut through the IOM ligament on one side of the jaw of 1-year-old adult zebrafish (Figures 1A–C). Compared to the uninjured fish in which pulling of the gill cover opens the jaw *via* the IOM ligament connection, pulling of the gill cover in the same fish post-injury fails to open the jaw, confirming a successful ligament transection (Supplementary Video S1). This failure to transmit force from the gill cover to the jaw is specific for the injured side, as the contralateral side displayed gill cover to jaw force transmission immediately upon recovery from anesthesia. Within 1 week after injury, pulling of the gill cover was able to open the jaw again, suggesting the rapid re-establishment of mechanical coupling between the gill cover and jaw joint (Supplementary Video S1). These observations demonstrate the feasibility of severing the IOM ligament and transiently decoupling and hence destabilizing the jaw joint from the supportive gill cover structure.

### Ligament Transection Induces Transient Degeneration Followed by Regeneration of Jaw Joint Cartilage

We next addressed the effect of IOM transection on jaw joint morphology in 1-year-old fish by performing a time course of histology following injury. We used a modified Osteoarthritis Research Society International (OARSI) scoring system to quantify the extent and severity of cartilage damage at the joint surface (Pritzker et al., 2006; Askary et al., 2016). In contrast to 1/29 jaw joints of uninjured controls having an OARSI score greater than 6 (representing mild damage), 11/31 jaw joints had an OARSI score greater than 6 at 14 days post-ligament transection (dplt) (Figures 1D–G). We also observed synovial hyperplasia on the same side of the jaw joint as the transected IOM in all 31 animals at 14 dplt (arrow in Figure 1E). Remarkably, we observed complete recovery of joint cartilage by 28 and 42 dplt, reflected by improvement in both the average OARSI score and the proportion of joints with OARSI >6 (2/13 at 28 dplt and 1/14 at 42 dplt) (Figures 1F,G). Despite the considerable variability in the degree of initial cartilage degeneration following IOM transection, our findings support the ability of damaged adult jaw joints in zebrafish to recover and replace missing joint cartilage within a few weeks after injury.

### Upregulation of Sox10 Expression and Cell Cycle Re-entry During Articular Cartilage Regeneration

**FIGURE 2:**
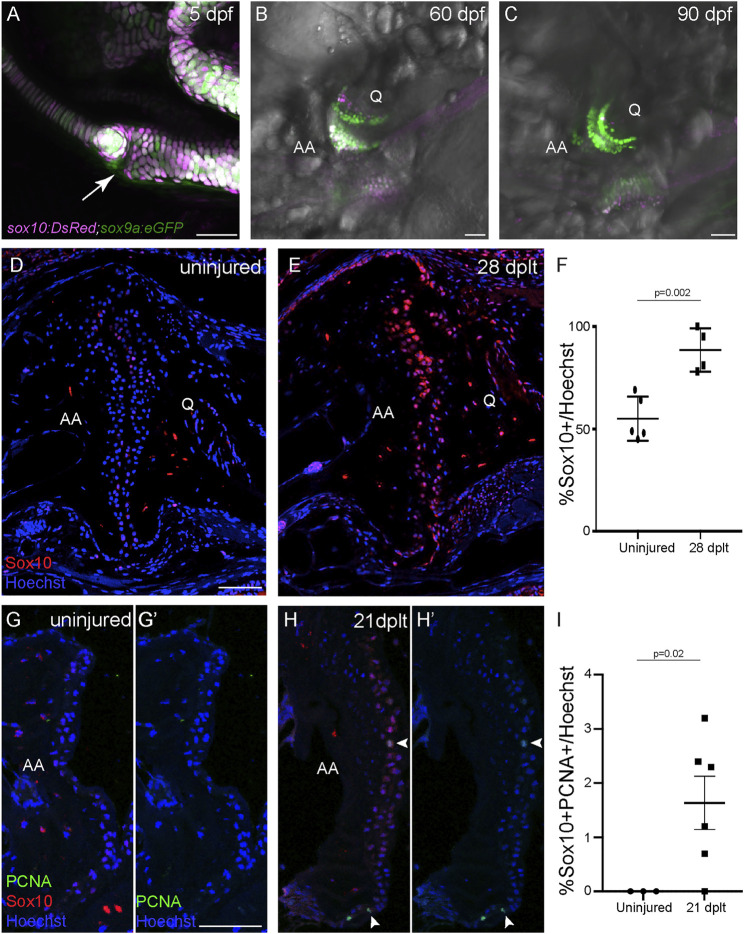
Sox10 expression is upregulated during cartilage regeneration. **(A–C)** *sox10:DsRed* and *sox9a:eGFP* are broadly co-expressed in chondrocytes at 5 days post-fertilization (dpf), and *sox10:DsRed* becomes restricted to a subset of joint chondrocytes at 60 and 90 dpf. The arrow shows the developing jaw joint at 5 dpf. **(D,E)** Antibody staining of the adult jaw joint shows endogenous Sox10 protein (red) in joint cells in 1-year-old uninjured control **(D)** and interopercular–mandibular (IOM)-transected animals at 28 days post-ligament transection (dplt) **(E)**. **(F)** Quantification of Sox10+ cells as a percentage of total Hoechst+ cells at the jaw joint articular surface. Individual data points, mean, and standard error of the mean are shown. The *p*-value is calculated by two-tailed Student’s *t*-test. **(G,H)** Antibody staining for PCNA (green) and Sox10 (red) of adult jaw joints from uninjured controls **(G,G′)** and IOM-transected animals at 21 dplt **(H,H′)**. The white arrowheads indicate double-positive PCNA+/Sox10+ nuclei. **(I)** Quantification of double-positive PCNA+/Sox10+ chondrocyte nuclei as a percentage of total Hoechst-stained nuclei at the jaw joint articular surface. Individual data points, mean, and standard error of the mean are shown. The *p*-value was calculated using Welch’s two-tailed *t*-test. AA, anguloarticular bone; Q, quadrate bone. Scale bars = 50 μm.

We next asked whether the embryonic cartilage differentiation program is reactivated during joint cartilage regeneration. SoxE factors (*sox9a* and *sox10* in zebrafish) are highly expressed during cartilage differentiation (Yan et al., 2005; Dutton et al., 2008), with Sox9 being particularly essential for cartilage formation across vertebrates (Bi et al., 1999; Mori-Akiyama et al., 2003; Yan et al., 2005). We confirmed the broad co-expression of *sox9a:GFP* and *sox10:DsRed* in the jaw chondrocytes of zebrafish at 5 days post-fertilization (dpf) (Figure 2A). In contrast, by 60 and 90 dpf, *sox9a:GFP* is maintained throughout the jaw joint chondrocytes, yet *sox10:DsRed* becomes restricted to a small subset of chondrocytes (Figures 2B,C). The Sox10 protein shows a similar restricted distribution in 1-year-old zebrafish, with a weak expression of Sox10 protein in about half of the chondrocytes at the jaw joint surface (Figure 2D). In contrast, 80–100% of chondrocytes expressed Sox10 protein in 1-year-old fish at 28 dplt, with the expression in individual chondrocytes being at much higher levels compared to the uninjured controls (Figures 2D–F). To assess the ability of Sox10+ cells to proliferate during joint cartilage regeneration, we performed co-immunostaining for Sox10 and proliferating cell nuclear antigen (PCNA) at 21 dplt. In the jaw joints of 1-year-old uninjured fish, we were unable to detect any Sox10+/PCNA+ cells at the articular surface (Figures 2G–I). In contrast, in regenerating animals at 21 dplt, an average of 1.6% of the total cells at the joint articular surface was Sox10+/PCNA+ (Figures 2H,I). These data indicate that a subset of cells re-enter the cell cycle and upregulate the embryonic cartilage gene *sox10* during adult joint cartilage regeneration in zebrafish.

### Sox10+ Cells Are Required for Regeneration of Jaw Joint Cartilage

**FIGURE 3:**
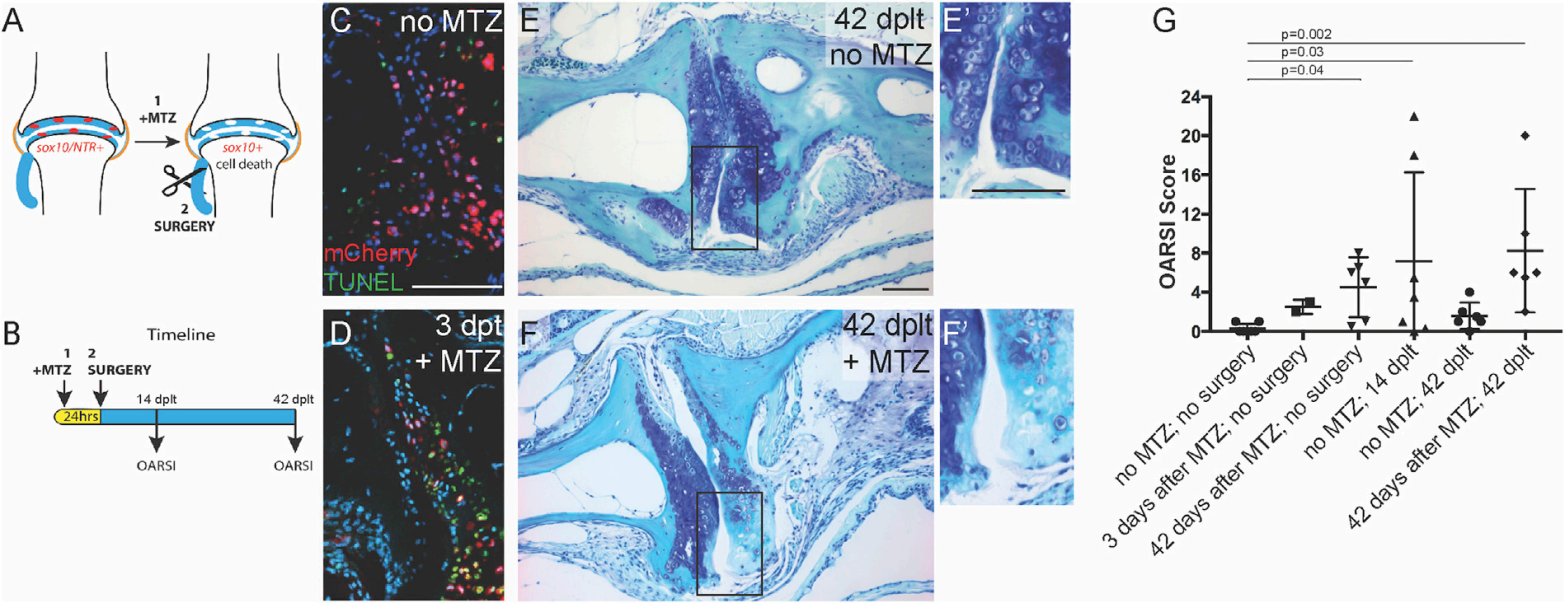
Sox10-expressing cells are required for cartilage regeneration. **(A)** Genetic ablation model. **(B)** Timeline for treatment and analysis. **(C,D)** Antibody and TUNEL staining of the adult jaw joint shows mCherry expression in *sox10:mCh-NTR* adult joint cells and apoptotic cells in metronidazole (Mtz)-treated animals at **(D)** 3 days after Mtz treatment (days post-ligament transection, dplt). **(E,F)** Toluidine blue staining of joints at 42 dplt without **(E)** or with Mtz treatment **(F)** prior to ligament injury (insets in **E′** and **F′** show the details of the articular surface). **(G)** OARSI scoring of joint integrity following Mtz treatment and/or ligament injury. Individual data points, mean, and standard error of the mean are shown. The *p*-values are calculated by Kruskal–Wallis ANOVA and Dunn’s multiple-comparison test. Scale bars = 50 μm.

In order to test the requirement of pre-existing Sox10+ chondrocytes for articular cartilage regeneration, we used a genetic strategy to selectively ablate Sox10+ chondrocytes prior to the joint-destabilizing injury (Figures 3A,B). To do so, we used *sox10:Gal4VP16* (Das and Crump, 2012) and *UAS:mCherry-NTR* (Davison et al., 2007) transgenic lines to drive the expression of a fusion between mCherry fluorescent protein and the bacterial nitroreductase enzyme (NTR) in Sox10+ chondrocytes. When the antibiotic metronidazole (Mtz) is delivered *via* the fish water, NTR converts Mtz into a cytotoxic agent, thus killing cells in a largely cell-autonomous manner (Curado et al., 2008). Compared to the dimethyl sulfoxide (DMSO)-treated controls, the treatment of 1-year-old *sox10>mCherry-NTR* fish overnight with 5 μM Mtz resulted in the effective killing of Sox10+ cells 3 days later as assessed by apoptotic TUNEL stain and loss of mCherry+ cells (Figures 3C,D). The ablation of Sox10+ cells resulted in a mild cartilage defect at 3 days post-treatment (mean OARSI, <4), which became slightly more pronounced at 42 days post-treatment (mean OARSI, <5) (Figure 3G). We then tested the effect on the regenerative response of ablating Sox10+ chondrocytes at 1 day prior to ligament transection. Compared to DMSO-treated controls that showed variable articular cartilage degradation at 14 dplt and recovery at 42 dplt, the Sox10-ablated animals displayed severe articular cartilage degradation at 42 dplt (mean OARSI, >8) (Figures 3E–G). These results show that the subset of chondrocytes that maintain Sox10 expression in the adult jaw joint is required for articular regeneration following injury-induced arthritis.

### Diverse Populations of Chondrocytes and Connective Tissue Cells are Present in Adult Zebrafish Jaw Joints

**FIGURE 4:**
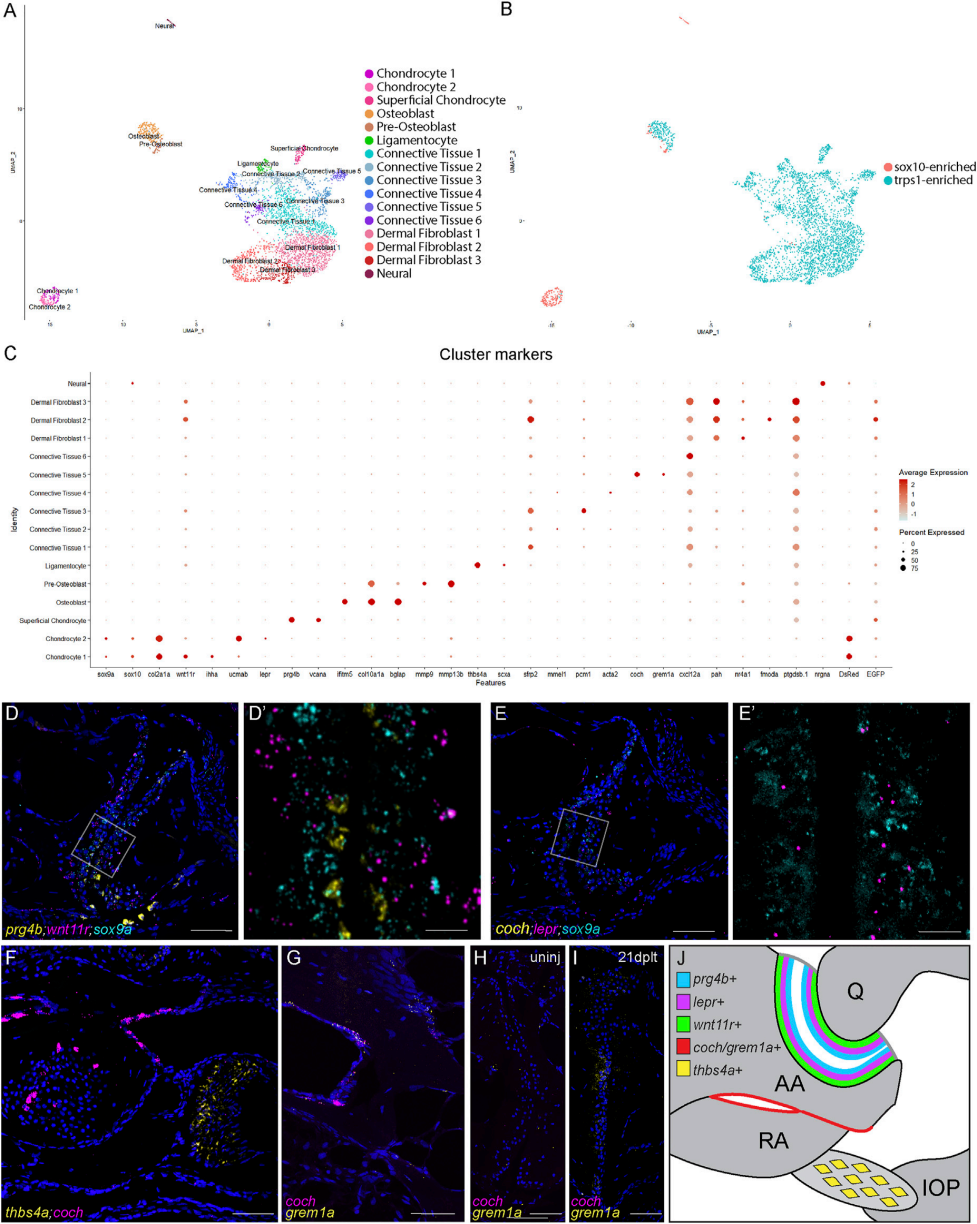
Single-cell transcriptome of adult zebrafish jaw joint. **(A, B)** UMAP visualization of scRNAseq analysis of FACS-sorted 3-month-old jaw joints. The colors were based on clustering **(A)** or library of origin **(B)**. **(C)** Dot plot showing the marker gene expression for each cell cluster as well as DsRed and GFP transcripts. **(D–G)** RNAscope *in situ* hybridization for markers for chondrocyte clusters **(D,E)**, for CT5 (*coch*) and ligament (*thbs4a*) **(F)**, and for CT5 (*coch* and *grem1a*) **(G)**. Nuclei stained with DAPI in blue. The magnified regions of the boxed regions **(D′,E′)** highlight different zones of joint chondrocytes and are shown without the DAPI channel. The section in **(F)** is above the jaw joint sections of **(D,E)**. **(H,I)** RNAscope *in situ* hybridization for *grem1a* and *coch* at the articular surface of uninjured **(H)** and 21 dplt **(I)** jaw joints. Nuclei stained with DAPI in blue. **(J)** Model of chondrocyte zones (blue, purple, and green), joint-adjacent periosteum (red), and interopercular–mandibular ligament (yellow) with marker genes in the adult jaw joint. Scale bars = 50 μm **(D–H)** and 10 μm **(D′,E′)**.

To understand the diversity of cell populations within the adult zebrafish jaw joint that could contribute to joint regeneration, we performed single-cell RNA sequencing of sorted cells expressing *trps1:GFP* and/or *sox10:DsRed* from 3-month-old jaw joints. Whereas *trps1:GFP* broadly labels mesenchymal cells and articular chondrocytes of the adult jaw joint, *sox10:DsRed* labels a subset of both articular and deeper chondrocytes (Askary et al., 2016). Using fluorescence-activated cell sorting of 50 pooled micro-dissected jaw joints, we separately isolated *sox10:DsRed*+ cells (both *trps1:GFP*-negative and -positive) and *trps1:GFP*+; *sox10:DsRed+* cells (Supplementary Figure S1). We then prepared two barcoded cDNA libraries using the 10X Genomics platform and performed Illumina next-generation sequencing. After filtering, we analyzed RNA expression in 4,149 total joint cells using the Seurat package in R (*sox10:DsRed*+, 237 cells; *trps1:GFP*+, 3,912 cells). Louvain clustering, followed by sub-clustering of osteoblast and chondrocyte populations, identified 16 cell clusters: neurons, two populations of osteoblasts, three populations of chondrocytes, ligamentocytes, six populations of connective tissue cells, and three populations of dermal fibroblasts (*pah*+, see bioRxiv 10.1101/2021.08.19.456710) (Figures 4A–C; Supplementary Table S1 for cluster cell counts separated by library; Supplementary Table S2 for cluster marker genes). The recovery of *GFP* and *DsRed* transcripts revealed *trps1:GFP* expression in all clusters, except neurons, and *sox10:DsRed* expression in chondrocytes, neurons, and a small number of osteoblasts (Figure 4B), largely consistent with previous *in vivo* imaging of these transgenic lines at the adult jaw joint (Askary et al., 2016).

Our analysis identifies three major chondrocyte clusters. Two of these are distinguished by high levels of *sox9a* and *sox10* expression as well as expression of typical cartilage matrix genes such as *col2a1a*. These *sox9a*+ chondrocytes include a cluster expressing mature (primarily pre-hypertrophic and hypertrophic) markers (*ihha* and *wnt11r*) and a cluster expressing immature markers (*ucmab* and *lepr*). The third major chondrocyte cluster expresses superficial-zone articular markers, including lubricin (*prg4b*), clusterin (*clu*), and versican (*vcana*). These superficial chondrocytes express higher levels of *trps1:GFP* but almost no *sox9a*, *sox10*, *sox10:DsRed*, or *col2a1a*, suggestive of a large-scale downregulation of cartilage matrix production. *In situ* hybridization confirmed distinct chondrocyte domains within 3-month-old jaw joints, with *prg4b* expression being high in superficial cells and *lepr* and *wnt11r* expression being high in successive layers of deeper chondrocytes co-expressing *sox9a* (Figures 4D,E). Interestingly, the superficial chondrocytes cluster more closely with ligamentocytes and other connective tissue clusters than with other chondrocytes, pointing to a strong fibroblast-like identity to these cells. The IOM ligamentocytes are marked by a high expression of *scxa* and *thbs4a* (Figures 4C,F). The sub-clustering of the osteoblast population reveals distinct pre-osteoblasts and osteoblasts. Pre-osteoblasts are defined by high levels of matrix metalloproteinases—in particular, *mmp9* and *mmp13b*—with a subset of these co-expressing *sox10:DsRed*. This finding is consistent with our previous studies showing that a subset of *mmp9*+ hypertrophic chondrocytes become osteoblasts in zebrafish bones (Giovannone et al., 2019).

Within the six connective tissue (CT) cell clusters, CT4 is enriched for *acta2* (smooth muscle actin) and likely represents a smooth muscle and/or pericyte population. CT1 and CT3 are characterized by the enrichment of the Wnt antagonist *sfrp2*, with CT3 selectively expressing pericentriolar material 1 (*pcm1*) that is involved in cilium assembly. CT6 is enriched for the chemokine *cxcl12a*, indicative of a stromal identity (Greenbaum et al., 2013). Of particular interest is CT5, which forms a distinct cluster from the other five and expresses the Bmp antagonist *grem1a* as well as *coch* that encodes a poorly understood extracellular matrix protein. *In situ* validation for *coch* and *grem1a* shows co-expression in a subset of periosteum adjacent to the jaw joint (Figures 4F,G). Whereas neither *coch* nor *grem1a* is expressed at the articular surface in uninjured one-year-old adult fish (Figures 4E–H), we observe the expression of *grem1a*, but not *coch*, at the articular surface during joint regeneration (*n* = 5/6 at 21 dplt; Figure 4I). Our data therefore reveal a remarkable diversity of joint-associated populations in the adult zebrafish jaw joint and point to a potential role of a joint-adjacent periosteal population in articular cartilage regeneration (Figure 4J).

## Discussion

Given the high prevalence of osteoarthritis in the adult population, new models of natural joint regeneration would be highly valuable in uncovering the capacity of tissue-resident progenitors for joint repair. Here we show that adult zebrafish display transient jaw joint degeneration in response to ligament transection, which is rapidly followed by the robust regeneration of missing joint cartilage and the re-establishment of normal joint architecture. One limitation of our joint injury model is the variability in degenerative response, with less than half of 1-year-old fish undergoing joint degeneration. This variability could reflect the natural resistance of zebrafish to joint cartilage loss and/or the high regenerative capacity of zebrafish, with new joint cartilage potentially being produced before full degenerative changes are apparent. Nonetheless, the ability to quantitatively assay many fish following joint-destabilizing injuries has allowed us to make new insights into the molecular and cellular basis of natural jaw joint regeneration.

During jaw joint regeneration, we observe a marked upregulation of Sox10 expression throughout joint chondrocytes. As in mammals, we find that adult articular chondrocytes are largely post-mitotic during homeostasis. However, in contrast to the lack of articular cartilage proliferation in rodent DMM arthritis models, at least some Sox10+ articular chondrocytes appear to re-enter the cell cycle in response to injury in zebrafish, which may contribute to the selective regenerative capacity of joint cartilage in fish. Sox-expressing chondrocytes were also recently described to regenerate a partial-thickness injury in adult skate (*Leucoraja erinacea*) (Marconi et al., 2020), suggesting that regenerative capacity is an ancestral feature of vertebrate cartilage. While Sox9 is the major SoxE factor required for chondrocyte differentiation (Bi et al., 1999), the related SoxE factor Sox10 is co-expressed during embryonic chondrogenesis, particularly in zebrafish (Dutton et al., 2008) but also, to a moderate extent, in mouse (Kaucka et al., 2017). During later development, *sox9a* expression is maintained in jaw joint cartilage, yet *sox10* expression is strongly downregulated, suggesting that Sox9a functions as the major maintenance factor of cartilage identity. Upregulation of *sox10* during regeneration could therefore reflect the reactivation of an embryonic chondrocyte differentiation program in new joint chondrocytes. As Sox9 has also recently been described to have a chondrocyte-protective function in load-bearing regions of articular cartilage in mice (Haseeb et al., 2021), *sox10* expression in chondrocytes that survive the destabilizing injury could also reflect increased SoxE dosage to protect the joint surface, potentially underlying the resistance to joint degeneration of a subset of 1-year-old fish. In the future, it will be important to develop labeling techniques to distinguish between newly generated and pre-existing chondrocytes as well as conditional loss-of-function strategies to remove *sox10* and/or *sox9a* prior to injury.

Our genetic ablation experiments show that the subset of joint chondrocytes that maintain *sox10* expression is essential for joint homeostasis and regeneration. Single-cell profiling reveals that *sox10*-expressing cells mainly consist of chondrocytes that are distinct from superficial *prg4b*+ chondrocytes. There are multiple possible interpretations of this finding. First, *sox10*-expressing cells could represent immature or embryonic chondrocytes that can rapidly proliferate and/or dedifferentiate to regenerate joint cartilage. Second, genetic ablation of a subset of deep-zone cartilage could further destabilize the joint, in addition to damage caused by ligament transection, beyond a threshold from which the zebrafish jaw joint can naturally recover. Third, there may be rare *sox10*-expressing non-chondrocyte populations that contribute to the repair. Teasing out these possibilities will require more specific lineage tracing tools in the future.

The single-cell analysis of the adult zebrafish jaw joint reveals similarities to mammalian joints—in particular, the presence of distinct deeper chondrocyte populations and superficial chondrocytes (Bian et al., 2020). Interestingly, we find that the transcriptome of superficial chondrocytes is more similar to ligamentocytes and connective tissue populations than deeper chondrocytes. Whereas it is likely that superficial chondrocytes derive from embryonic chondrocytes in zebrafish, their distinct transcriptome may reflect a strong downregulation of cartilage matrix gene expression and a shift to a more specialized expression of synovial genes such as *prg4b*. We also identified six distinct connective tissue populations that would be interesting to test for their roles in regeneration. These include *cxcl12a*+ stromal cells (CT6), reminiscent of *Cxc12*+ bone marrow stromal cells that contribute to new bone during fracture repair (Matsushita et al., 2020), and two populations (CT1 and CT3) marked by the expression of the *sfrp2* inhibitor of Wnt signaling, a pathway known to play an important role in joint development and disease (Luyten et al., 2009). Another population is characterized by the expression of *coch* and *grem1a* (CT5), with *in situ* validation showing that it represents a joint-adjacent periosteal cell population. In mice, Grem1 has been shown to mark skeletal progenitors in embryonic bones (Worthley et al., 2015) and a subset of synovial cells and articular chondrocytes in the knee joint (Roelofs et al., 2020). Interestingly, we observe an expression of *grem1a* in articular chondrocytes during jaw joint regeneration. This could reflect perdurance of *grem1a* transcripts and contribution of CT5 periosteal cells to new Sox10+ articular chondrocytes during regeneration and/or upregulation of *grem1a* expression in newly formed or pre-existing articular chondrocytes. In the future, lineage tracing of distinct CT populations during joint regeneration will inform the extent to which contributions from resident CT progenitor populations *versus* proliferation of pre-existing chondrocytes underlie the ability of zebrafish, but not mammals, to regenerate synovial joints.

## Materials and Methods

### Zebrafish Lines

Published zebrafish lines include *Tg*(*sox10:DsRed*)^*el10*^ and *Tg*(*sox10*:*Gal4VP16*)^*el159*^ (Das and Crump, 2012), *Tg*(*trps1:EGFP*)^*j127aGt*^ (Talbot et al., 2010), *Tg*(*UAS-E1B:NTR-mCherry*)^*c264*^ (Davison et al., 2007), and *Tg*(*sox9a:GFP*)^*zc81tg*^ (Talbot et al., 2016).

### IOM Surgery

IOM ligament transection was performed on 8–12 months post-fertilization adult zebrafish using 3-mm Vannas spring scissors (Fine Science Tools, cat. #1500000). The fish were anesthetized using MS222 and placed ventral side up on a wet sponge, and a single cut was performed to transect the ligament. Transection was confirmed by manual pulling of the IOP bone.

### Histology, *In Situ* Hybridization, and Immunohistochemistry

Adult zebrafish were processed for paraffin embedding and histological analysis as previously described (Askary et al., 2016). Briefly, the heads were fixed overnight at 4°C in 4% paraformaldehyde, washed 2× in phosphate-buffered saline with Tween® 20 (PBST) for 30 min, and decalcified in 20% EDTA for 10 days rocking at room temperature (RT). The tissue was dehydrated through an ethanol and Hemo-De series and then embedded in paraffin. Next, 5-μm paraffin sections were cut on a Leica RM2235 or Thermo Scientific HM355 S microtome. Toluidine blue staining was performed on deparaffinized and rehydrated 5-μm sections. The slides were incubated for 10 min in 0.04% Toluidine Blue (Sigma, 89640) in 0.1 M sodium acetate, pH 4.0, followed by 3 × 1-min washes in water, incubation in 0.1% Fast Green FCF (Fisher Reagents, BP123-10) in water for 3 min, followed by 3 × 1-min washes in water, and mounting with cytoseal. For Sox10 immunohistochemistry, the slides were deparaffinized in xylene and then washed in PBST. Antigen retrieval was performed for 7 min in acetone at −20°C, followed by blocking in 2% goat serum in PBST for 30 min and incubation overnight at 4°C in primary rabbit anti-Sox10 antibody (1:250, Genetex, cat. #GTX128374). The slides were washed in PBST, then incubated for 1 h at RT in secondary antibody goat anti-rabbit Alexa Fluor 568 (1:500, ThermoFisher) diluted in blocking buffer and Hoechst 33342 nuclear stain (1:1,000, Fisher Scientific, 51–17), and mounted with Fluoromount-G (SouthernBiotech, 0100–01). For Sox10 and PCNA immunofluorescence, slides of 5-µm tissue sections were deparaffinized in xylene and ethanol and then washed in PBST. Antigen retrieval was performed for 35 min in 0.1 M sodium citrate buffer, pH 6, in a steamer at 89°C, followed by 30 min of blocking in 2% goat serum and 1% DMSO in PBST. The slides were washed in PBST and then incubated overnight at 4°C with primary rabbit anti-Sox10 and primary mouse anti-PCNA (1:1,000, Sigma-Aldrich, Cat# P8825) in blocking buffer. The slides were then washed in PBST, incubated for 1.5 h in secondary antibody goat anti-rabbit Alexa Fluor 568 (1:500, ThermoFisher) and goat anti-mouse Alexa Fluor 488 (1:500, ThermoFisher) with Hoechst 33342 nuclear stain, and then mounted with Fluoromount-G. Single-molecule fluorescence *in situ* hybridization was performed according to manufacturer guidelines using RNAScope Multiplex Fluorescent Reagent Kit v2 Assay (ACD Bio). Probes for *lepr* (C1), *sox9a* (C3), *wnt11r* (C1), *prg4b* (C2), *coch* (C2), *grem1a* (C1), and *thbs4a* (C1) were synthesized by Advanced Cell Diagnostics. Opal 520, 570, and 690 fluorophore reagents were used to visualize the expressions (Akoya Biosciences, cat. #FP1487001KT, #FP1488001KT, and #FP1497001KT). The sections were counterstained with DAPI and mounted with Fluoromount-G. Fluorescence imaging of tissue sections and live animals was performed using a Zeiss LSM800 or Leica SP8 confocal microscope. Histological slides were imaged using a Leica D8 2500 microscope. All imaging settings were modified consistently across samples during imaging and during image processing in Adobe Photoshop for each experiment.

### Drug Treatments

Cell ablation was carried out with an overnight treatment of 5 μM metronidazole (MTZ, Sigma-Aldrich, M1547) in tank water. The fish were then placed in fresh system water to recover. mCherry fluorescence was assessed prior to and after treatment to confirm cell ablation. Apoptosis was also assessed in 5-μm tissue sections through the TUNEL method using the ApopTag Fluorescein *In Situ* Apoptosis Detection Kit (Sigma-Aldrich, S7110) as per the instructions of the manufacturer with co-labeling using primary mouse anti-mCherry antibody (1:200, Novus Biologicals, NBP1-96752), secondary goat anti-mouse Alexa Fluor 568 (1:500, ThermoFisher), and DAPI nuclear stain.

### Single-Cell RNA Sequencing

#### Library Preparation and Alignment

A total of 50 micro-dissected jaw joints from *sox10:DsRed*+; *trps1:GFP*+ transgenic animals were mechanically and enzymatically dissociated into a single-cell suspension. The joints were incubated in protease solution [0.25% trypsin (Life Technologies, 15090–046), 1 mM EDTA, and 400 mg/ml collagenase D (Sigma-Aldrich, 11088882001) in PBS] at 28.5°C and agitated with a P1000 pipet every 5 min for 1.5 h until fully dissociated. The cells were pelleted (5 min, 2,000 rpm, 4°C) and washed once in suspension media [1% fetal bovine serum, 0.8 mM CaCl_2_, 50 U/ml penicillin, and 0.05 mg/ml streptomycin (Sigma-Aldrich) in phenol red-free Leibovitz’s L15 medium (Life Technologies)] before resuspension and pooling into a final volume of 500 ul. Single cells were sorted by fluorescence-activated cell sorting to isolate DsRed-positive and GFP-positive populations into PBS containing 0.1% BSA. Single-cell RNA sequencing library construction was performed as per the instructions of the manufacturer using 10X Genomics Chromium Single Cell 3′ Library and Gel Bead Kit v.2. Libraries were sequenced using the Illumina NextSeq at a depth of 131,341 mean reads per cell (GFP), and 151,840 mean reads per cell (DsRed). Cellranger v3.0.0 (10X Genomics) was used for alignment to GRCz11 built with v4.3.2. gtf (Lawson et al., 2020), including GFP and DsRed sequences as additional artificial chromosomes to generate gene-by-cell count matrix generation with default parameters.

#### Processing and Analysis of scRNAseq Data

Analyses of scRNAseq libraries were performed using R and RStudio with Seurat version 4 (Hao et al., 2021). Datasets from *sox10*-enriched and *trps1*-enriched samples were first merged into one object. Cells with <500 or >2,000 unique genes expressed or >5% mitochondrial RNA were excluded to remove low-quality, doublet, or stressed and dying cells. Additionally, epithelial cells (*epcam+*) and endothelial cells (*pecam1+*) were removed from the downstream analysis. The data were log-normalized and scaled, and variable features were identified using the default parameters with regression for percent mitochondrial RNA in scaling. Linear dimensional reduction was then performed using the RunPCA function for principal component analysis (PCA) on 50 principal components. Performing a jackstraw on the PCA identified 38 significant dimensions (*p* < 0.05) which were included in the downstream analysis. Clusters were determined using functions FindNeighbors for dimensions 1–38 and FindClusters using a resolution of 0.9. The osteoblast cluster (enriched for *col10a1a*) and chondrocyte cluster (labeled by *col2a1a* and *mia*) were then subclustered into 2 subclusters, each using function FindSubCluster with a resolution of 0.2 and 0.5, respectively. Clusters and gene expression were visualized using UMAP non-linear dimensional reduction. Function FindAllMarkers was used to identify the markers of each cluster and subcluster relative to the rest of the dataset using a log fold change threshold of ≥0.2.

### Quantification and Statistical Analysis

Experimental and control zebrafish for each experiment were age- and stage-matched using standard body length (Parichy et al., 2009). Two independent researchers performed blinded OARSI scoring on 3–5 representative toluidine blue-stained images for each scored joint for all experiments (as per Askary et al., 2016). The OARSI scores for zebrafish joints were compared by ANOVA and Tukey’s multiple-comparison test (Figure 1) and Kruskal–Wallis ANOVA test with Dunn’s multiple-comparisons test (Figure 3) to determine significance between groups using GraphPad Prism 8 and 9. The Sox10+ cell counts in adult joint cartilage were assessed by two-tailed Student’s *t*-test in GraphPad Prism 8 and graphically represented as individual counts, mean, and standard error of the mean for each condition. The Sox10+/PCNA+ cell counts in jaw joint articular cells were quantified as a proportion of total Hoescht-stained nuclei by researchers blinded to the treatment. Percentages of Sox10+/PCNA+ nuclei were analyzed with Welch’s *t*-test in GraphPad Prism 9, graphically represented as individual counts, mean, and standard error of the mean for each condition.

## Data Availability

The original contributions presented in the study are publicly available. These data can be found here: National Center for Biotechnology Information (NCBI) BioProject database under accession number GSE184403.
