# Supplementary figures and images for: Regeneration of Jaw Joint Cartilage in Adult Zebrafish
Source: Front Cell Dev Biol. 2022 Jan 20;9:777787. doi: 10.3389/fcell.2021.777787 (PMC8811260; doi:10.3389/fcell.2021.777787)

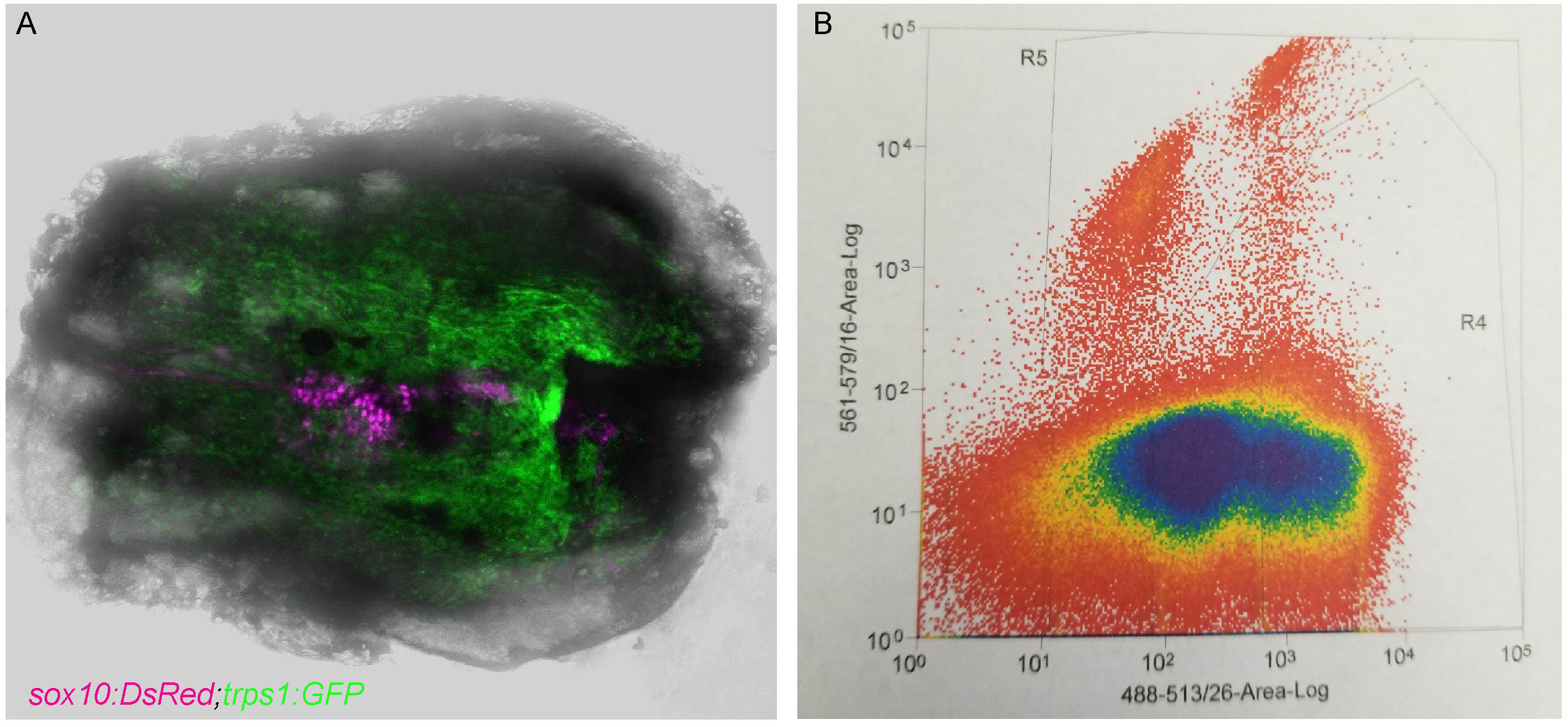

Supplement: Supplementary file 1 [file Image1.TIF]
